# Correspondence

**Published:** 1872-07

**Authors:** J. D. Patterson

**Affiliations:** Lawrence


### CORRESPONDENCE.

LAWRENCE, June 6, 1872.

DR. J. TAFT, *Editor Dental Register:*

In Dr. Driscoll's article, "Unnecessary and Unkind," which appears in the May No. of the REGISTER, the writer does me injustice by giving as my treatment for Alveolar Abscess, a few sentences I wrote as to treatment of extreme cases, "*where the discharge could not be stopped in the usual way*;" and thus endeavoring to instruct the reader that such was my *usual method.* Now I stated my general treatment before giving this which I sometimes used in bad cases, and it does not differ materially from that of Dr. McDonneld, which Dr. Driscoll gives in comparison; but Dr. Driscoll ungenerously takes the *tail end* of my remarks on abscess, and compares with Dr- Mc. Donneed's method.

I desire to protest against such procedure. I cannot see why Dr. Driscoll should endeavor to take such advantage. As to other criticisms I do not feel called on to say anything, as no one will be benefitted by quibbling over unimportant items, such as my giving incorrect dates, etc.

With Respect, Yours Truly,

J. D. PATTERSON,

